# Electrochemical Determination of the Antioxidant Potential of Some Less Common Fruit Species

**DOI:** 10.3390/s8127564

**Published:** 2008-11-26

**Authors:** Zbynek Gazdik, Boris Krska, Vojtech Adam, Jan Saloun, Tunde Pokorna, Vojtech Reznicek, Ales Horna, Rene Kizek

**Affiliations:** 1 Department of Breeding and Propagation of Horticultural Plants; Zemedelska 1, CZ-613 00 Brno, Czech Republic; 2 Department of Agrochemistry, Soil Science, Microbiology and Plant Nutrition; Zemedelska 1, CZ-613 00 Brno, Czech Republic; 3 Department of Chemistry and Biochemistry, Zemedelska 1, CZ-613 00 Brno, Czech Republic; 4 Department of Fruit Growing, Faculty of Horticulture, Mendel University of Agriculture and Forestry, Valtická 337, CZ-691 44 Lednice, Czech Republic; 5 Department of Animal Nutrition and Forage Production, Faculty of Agronomy, Mendel University of Agriculture and Forestry, Zemedelska 1, CZ-613 00 Brno, Czech Republic; 6 Department of Applied Pharmacy, Faculty of Pharmacy, University of Veterinary and Pharmaceutical Sciences, Palackeho 1 - 3, CZ-612 42 Brno, Czech Republic; 7 Institute of Natural and Informatics' Sciences, Faculty of Central European Studies, Constantine the Philosopher University in Nitra, Nabrezie mladeze 91, SK-949 76 Nitra, Slovak Republic; 8 Tomas Bata University, T.G. Masaryka 275, CZ-762 72 Zlin, Czech Republic

**Keywords:** Less Common Fruit Species, Flavonoids, Antioxidant Capacity, Liquid Chromatography with Electrochemical Detection, DPPH• test

## Abstract

Various berries and fruit types of less common fruit species are known to contain antioxidants. Consumption of high amounts of antioxidant flavonoids, which display a variety of biological properties, including antiproliferative and anti-inflammatory activity, may have a positive impact on human health, particularly for the prevention of cancer and other inflammatory diseases. In these studies, based on the hypothesis that the fruit extract with the highest content would possess significantly higher health benefits, flavonoid-rich extracts were obtained from some less common fruit species – Blue Honeysuckles (*Lonicera Kamtschatica* and *Lonicera edulis*, Turcz. ex. Freyn), Saskatoon berry (*Amelanchier alnifolia* Nutt.) and Chinese Hawthorn (*Crataegus pinnatifida* BUNGE) – grown from germplasm held at the Mendel University of Agriculture and Forestry in Brno, Czech Republic and then characterized in terms of biological value based on the results from a relative antioxidant capacity assessment. The antioxidant content evaluation was based on the total flavonoid amount, determined by liquid chromatography with electrochemical detection (HPLC-ED). A DPPH• test was applied as a reference. The antioxidant content measured in Chinese Hawthorn fruit extract identified it as a potent source of flavonoid antioxidants, with a content 9-fold higher than that seen in *Amelanchier* fruit. The multifunctional HPLC-ED array method coupled with a DPPH• reference appears to be the optimal analytical progress, accurately reflecting the nutritive-therapeutic properties of a fruit.

## 1. Introduction

Fruits and vegetables are examples of a dietetically important group of foodstuffs. These components of human diet are not adequately replaceable by any other products. Plant tissues are naturally rich in nutritive or therapeutically active products of plant secondary metabolism. The consumption of fruits and vegetables has been inversely associated with morbidity and mortality from degenerative diseases [1-3]. It is not known which dietary constituents are responsible for this association, but antioxidants appear to play the major role in the protective effects of plant foods [4-8]. Less common fruit species such as Blue Honeysuckle, Saskatoon berry, Chinese Hawthorn etc. are assumed to provide fruits with high biological value. Given the simplicity of their growth, they can now be considered agricultural human diet commodities. In order to correctly identify less common fruit species that might merit consideration as functional foodstuffs, identification of the biologically active substances they may contain is necessary. The evaluation of the berries of less common fruit species is difficult because typically their ingredients are not completely known. Due to the chemical diversity of antioxidant compounds present in foods, complete databases on food antioxidant content are not yet available. In addition, levels of single antioxidants in food do not necessarily reflect their total antioxidant capacity [2]. The main problem in this area is caused by the shortcomings of standard analytical methods and techniques for the identification of these substances in complex matrices such as plant tissues. Due to this, often only a particular group of substances or more functionally connected indicators of biological value are evaluated. Nevertheless, a more complex point of view on total oxidant capacity (TOC) should be considered. Determination of antioxidant activity is one of the possibilities available for expressing the biological value of the foodstuff, concurrently with the assessment of the main bioactive components represented in the berries. It is known that substances without electrochemical activity show reduced antioxidant activity. On the contrary, in the substances with low half-wave oxidant potential, the ability of inactivation of free oxygen radical forms is probable [9, 10]. This work was focused on the suggestion that electroanalytic determination of total antioxidant capacity might be a useful tool to compare the biological value of berries in the chosen less common fruit species.

## 2. Results and Discussion

The antioxidative activity of plant tissue functional substances plays an important role amongst the positive biological effects of foodstuffs on human health and welfare. Because plants contain many different classes and types of antioxidants, knowledge of their total antioxidant capacity (TAC), which is the cumulative capacity of food components to scavenge free radicals, would be useful for epidemiological purposes [1, 3, 4, 11, 12]. In accordance with the investigated protective effects of several plant foods, TAC is thought to be one of the basic measures of a foodstuff's biological value. Several methods have been developed for measuring the total antioxidant capacity of foods and beverages [13, 14]. High performance liquid chromatography coupled with an electrochemical multichannel detector (HPLC-ED) has been utilized for determination of the content of bioactive phenols. Electrochemical analysis using liquid chromatography of the total content of antioxidative active phenolic acids and polyhydroxylated derivatives of flavan was the technique selected for TAC determination in the extracts of the berries of less common fruit species. Peaks showing detected antioxidant complex(es) were summarized and their areas were then integrated (derived) to obtain the relative values. Obtained data (%) were interpreted as relative antioxidant capacity [15].

**Figure 1.**
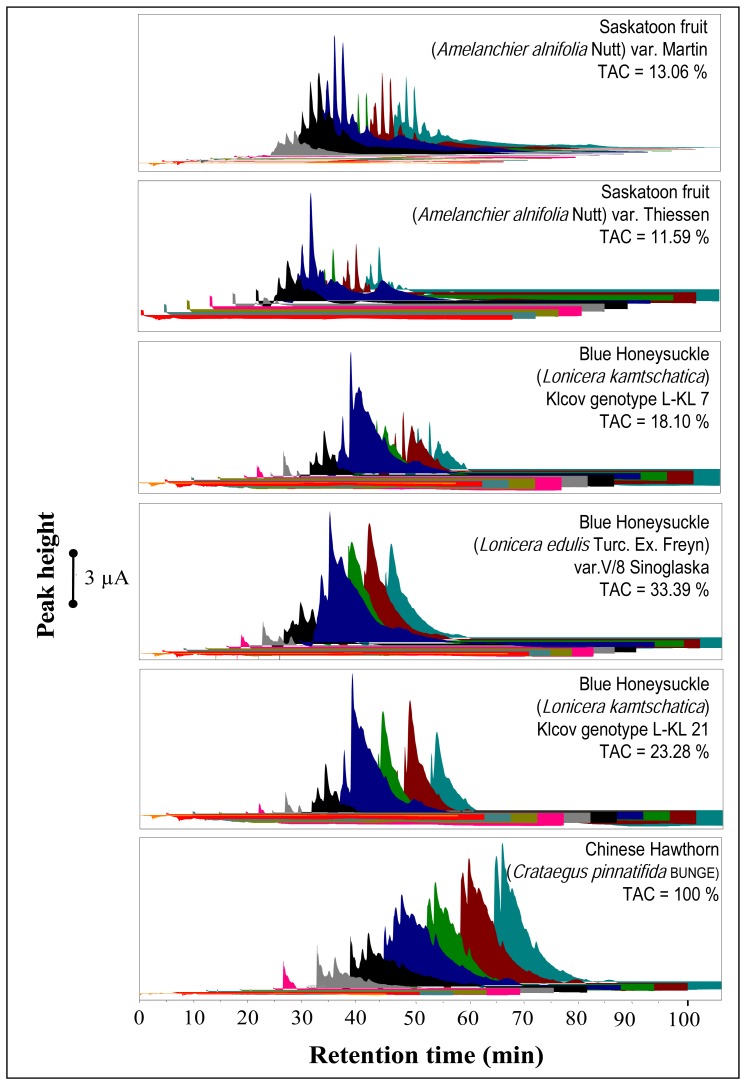
HPLC-ED chromatograms of fruit samples. The potentials applied on the electrode were -80, 0, 80, 160, 240, 320, 400, 480, 560, 640, 720 and 800 mV. Other experimental conditions see in “Material and Methods” section.

Because different antioxidant compounds may act *in vivo* via different mechanisms, no single method can fully evaluate the TAC of foods [11, 16]. The reference method used for the verification of the results obtained from the HPLC-ED analysis was the DPPH• test. Among the fruits such as apricots or peaches belonging to the *Rosaceae*, Hawthorn fruit (*Crataegus pinnatifida* Bunge), whose analytic profile has not been compiled yet, showed the highest antioxidant capacity by both methods (Figure 1). This observation is in good agreement with the higher phenolic content of Hawthorn fruit compared to other stone fruits rpdiuced by the *Rosaceae* described by Ercisli [17]. A relatively high level of antioxidative capacity was found in the extracts from Honeysuckle berries (*Lonicera edulis*, Turcz. ex. Freyn). Such a high level of antioxidant capacity is in agreement with the literature and is likely due to the high content of phenolic acids and flavonoids [16], which were discovered in the berries of different taxons of the genus *Lonicera* L. The sequence of berries in the graphs, drawn on the basis of the results from HPLC-ED and DPPH^·^ tests, was different in the two samples (Figure 1). The different sequence in the graph of DPPH• test, in comparison with the sequence in the graph of HPLC-ED, was discovered in the sample of Shadbush var. Martin and the L-KL-21 Honeysuckle genotype (Figure 1). The difference in the sequences, compiled on the basis of two different methods of evaluation of biological value, reflects high content of antioxidants with the absence of phenol rings in the extracts of Shadbush var. Martin. In such cases, phenolic components, which have demonstrated strong antioxidant activities in different model systems [15, 18] do not represent the major antioxidant complex. However, even if different rankings of TAC were reported among these fruits, there is agreement in the literature that Amelanchier fruit is one of the most effective of functional foods.

To verify correlation among methods, Pearson's correlation analysis was performed using statistical software (*UNISTAT*); *P*-values < 0.05 were considered significant. Statistic evaluation of the effective determination of total antioxidative capacity by Pearson method applied on the results of HPLC-ED/DPPH^·^ shown that the incorporation of electrochemical analyses with a suitable referential method can lead to increase predictive value of an assay. In the level of statistical importance *P* < 0.05, both applied procedures seem to be equivalent, correlation coefficient was 0.97.

## 3. Material and Methods

### 3.1 Chemicals

2,2-diphenyl-1-picrylhydrazyl DEPTH^•^, liquid nitrogen, 96% ethanol (Dr. Kulich Parma Czech Rep.), 80% methanol (Sigma Aldrich, Chemical Corp. St. Louis, USA), acetonitrile for HPLC (Sigma Aldrich, Chemical Corp. St. Louis, USA), other chemical substances used (Sigma Aldrich, Chemical Corp. St. Louis, USA) were of ACS purity.

### 3.2 Instruments

Automatic spectrometric analyzer BS-200 (Mindray, China), centrifuge (Eppendorf 5804R, Germany) and microfilters 0.45 μm with Teflon membrane (Metachem, Torrance, CA, USA) were used in our experiments. HPLC-ED chromatographic system consisted of DGU-20As vacuum degasser (Shimadzu Corp., Japan), two Model 582 ESA chromatographic pumps (ESA Inc., Chelmsford, MA, USA), a Zorbax C18-AAA (150×4.6 mm, 3.5 μm particle size, Aglient Technologies, USA) reverse phase chromatography column, a control module (Model 5600A, ESA, USA) equipped with three flow cells (Model 6210, ESA, USA), gradient mixer and repulser. Each cell consists of four analytical cells. One analytic cell contains a working carbon porous electrode, two auxiliary and two reference electrodes. Both the detector and column were thermostated at 30 °C. A sample was injected using autosampler (Model 540 Microtiter HPLC, ESA, USA). The data obtained were processed with the CoulArray Data Station Program 3.01 (ESA, USA).

HPLC-ED conditions were as follows: volume of injected sample was 30 μL. A flow rate of the mobile phase consisted from 0.2% (v/v) formic acid and acetonitrile was 0.35 mL min^-1^. A gradient profile starting at 12:88 (v/v, formic acid: acetonitrile) was increasing to 22:78 during first twenty minutes, then increasing to 50:50 during 5 minutes, then increasing to 55:45 during 5 minutes and finally decreasing linearly up to 15:85 from 30 to 40 min.

### 3.3 Fruit samples originated from less common fruit species

Three genotypes of Blue Honeysuckles: i) *Lonicera edulis*, Turcz. Ex. Freyn, V/8 Sinoglaska; ii) *Lonicera Kamtschatica*, L-KL-21 and iii) *Lonicera Kamtschatica*, L-KL-7, two varieties of Saskatoon berry (*Amelanchier alnifolia* Nutt.): i) var. Martin and ii) var. Thiessen and Chinese Hawthorn (*Crateagus pinnatifida* Bunge) were used in our experiments. Fruits of Blue Honeysuckles genotypes were harvested in mid-May 2007 and stored at -18 °C for eight months prior to analysis. Fruits of Saskatoon varieties were harvested during July 2007 and stored at -18 °C for six months prior to analysis. Chinese Hawthorn fruits were harvested in the beginning of October 2007 and stored at -18 °C for three months prior to analysis.

### 3.4 Sample preparation

Fruits were homogenized in the dark room at 4 °C. Weighed fruits (app. 5 g) were transferred to mortar, and liquid nitrogen was added. The frozen sample was grinding for 5 min. Then 10 ml of 80% methanol (prior to HPLC) or 10 ml of 90% ethanol (prior spectrometric analysis) was added to the mortar, and the sample was grinding for 10 min. The homogenate was transferred to test-glass. The mixture was homogenized by shaking on a Vortex-2 Genie (Scientific Industries, New York, USA) at 4 °C for 30 min. The homogenate was centrifuged (14.000 g) for 30 min at 4 °C using a Universal 32 R centrifuge (Hettich-Zentrifugen GmbH, Tuttlingen, Germany). Before the analysis the supernatant was filtered through a membrane screen disc (0.45 μm Nylon filter disk, Millipore, Billerica, Mass., USA). Filtrate (150 μl) was filled up with 150 μl 80% methanol or 90% ethanol.

## 4. Conclusions

The sensitivity and selectivity of these procedures is higher in comparison with the methods using conventional principles. Analytic evaluation of antioxidative capacity in foodstuff based on accurate quantification of majority antioxidants has high predictive value. Thanks to ED analyses it is possible to observe reactive kinetics of antioxidants in applied potentials, evaluate the structure of antioxidative complexes and their concern in total antioxidative capacity, whose different functional basis was determined in examined samples in this work.
